# Occurrence, distribution, and health risk assessment of quinolone antibiotics in water, sediment, and fish species of Qingshitan reservoir, South China

**DOI:** 10.1038/s41598-020-72324-9

**Published:** 2020-09-25

**Authors:** Liangliang Huang, Yuanmin Mo, Zhiqiang Wu, Saeed Rad, Xiaohong Song, Honghu Zeng, Safdar Bashir, Bin Kang, Zhongbing Chen

**Affiliations:** 1 College of Environmental Science and Engineering, Guilin University of Technology, Guilin, 541004 China; 2 School of Environment and Energy, South China University of Technology, Guangzhou, 510641 China; 3 Guangxi Key Laboratory of Environmental Pollution Control and Technology, Guilin University of Technology, Guilin, 541004 China; 4 Coordinated Innovation Center of Water Pollution Control and Water Security in Karst Area, Guilin University of Technology, Guilin, 541004 China; 5 Sub-Campus Depalor Okara, University of Agriculture Faisalabad, Okara, 56130 Pakistan; 6 Fisheries College, Ocean University of China, Qingdao, 266003 China; 7 Department of Applied Ecology, Faculty of Environmental Sciences, Czech University of Life Sciences Prague, Kamýcká 129, 16500 Prague, Czech Republic

**Keywords:** Ecology, Environmental sciences

## Abstract

The residual antibiotics in the environment have lately caused widespread concerns. However, little information is available on the antibiotic bioaccumulation and its health risk in drinking water resources of South China. Therefore, the occurrence, distribution, and health risk of four quinolone antibiotics including ofloxacin (OFX), norfloxacin (NOR), ciprofloxacin (CIP), and enrofloxacin (ENR) in the Qingshitan reservoir using high-performance liquid chromatography were investigated. Results revealed that the concentrations in water, sediment, and edible fish ranged from 3.49–660.13 ng/L, 1.03–722.18 μg/kg, and 6.73–968.66 μg/kg, respectively. The ecological risk assessment via the risk quotient (RQ) method showed that the values in sediment were all greater than 1, posing a high risk to the environment. The health risk index of water samples was at the maximum acceptable level, with OFX at the top while the rest were at the medium risk level. The main edible fish kinds of the reservoir had high dietary safety and the highest contaminations were found in carnivorous feeding habits and demersal habitat fishes with OFX as the highest magnitude. Source identification and correlation analysis using SPSS showed significant relationships between NOR with pH and turbidity (in water), as well as total phosphor (TP) and total organic carbon (TOC) in sediment. NOR was the highest in sediment which mostly sourced from livestock wastewater, croplands irrigation drain water, and stormwater. Correlations between CIP and ENR with TP were significant, while OFX was positively associated with total nitrogen (TN) which mainly originated from urban sewage as well as directly dosed drugs in fish farms. In conclusion, our results are of great significance for ensuring the safety of drinking water and aquatic products in this region.

## Introduction

Antibiotics, such as chloramphenicol, tetracyclines, sulfonamides, macrolides, quinolones, and beta-lactams are a class of secondary metabolites, produced by microbial organisms (including bacteria, fungi, and actinomycetes) or some plants and animals, that have anti-pathogens or other competences^1^. They are widely used in medicine, agriculture, animal husbandry, and aquaculture as an effective drug for treating diseases and promoting growth^2^, among which quinolones are the most frequently used type owing to their characteristics of broad-spectrum, high efficiency, easy to use and few adverse reactions. As a consequence, those antibiotics will enter water bodies through humans, livestock, aquaculture and so on.

In recent years, different levels of antibiotics have been reported in sediments and aquatic products in various regions. Wu et al.^3^ studied antibiotic contamination in the sediments of the Yongjiang river (Nanning City section), and detected antibiotics including methoxycinaxime, sulfonamides, and macrolides with the concentrations ranged from 1.08 to 30.84 μg/kg. Antibiotics were also detected in sediments of the suburbs of Tianjin. The detection rates of sulfamethoxazole, sulfamethizole, sulfadimethoxine, and norfloxacin were high, with concentrations ranging from 1.5 to 30.1 μg/kg^4^. Antibiotics were also found in other regions such as the Pearl River estuary^5^, Huangpu river^6^, Dalian offshore^7^, and Three Gorges reservoir area^8^. Yang et al.^9^ reported residues of quinolones in fish in Guangzhou. Antibiotics were detected in aquatic products of Ningbo, Beijing, Shanghai^10^, India^11^, Vietnam^12^, South Carolina, and Georgia^13^. China is not only a major producer of antibiotics but also a major user^14,15^. According to statistics, there are about 750 ~ 1,000 t chlortetracycline, 500 ~ 700 t oxytetracycline, 400 t norfloxacin, 100 t ciprofloxacin, and 15 t ofloxacin used for animal feed additives each year. In 2003, the veterinary drug use of thiamphenicol and florfenicol in China was approximately 400 t, while the total production of sulfonamides exceeded 20,000 t, of which about 4,000 t were used as veterinary medicine in China^16^. In 2013, the total amount of antibiotics used in China was approximately 162,000 t, of which 48% were for human use and the rest were for veterinary antibiotics^17^.

With the rapid development of the economy, a large number of synthetic compounds continue to enter the environment through production, transmission, and use, posing a potential threat to the ecosystems. Therefore, risk assessment of pollutants is an urgent task and an important part of the environmental management decision-making process. It is also the inevitable result of environmental science development^18^. Since 1993, the United States and the European countries have gradually begun to conduct drug risk assessments^19^. It refers to an assessment of adverse ecological impacts caused by one or more internal or external factors and can predict future ecological adverse effects or assess the likelihood of ecological changes due to certain factors in the past.

Qingshitan reservoir was listed as the second-level surface water source protection zone for drinking water by Guangxi region government in 2012. Studies on Qingshitan reservoir have mainly focused on water quality^20^, runoff change^21^, sediment organochlorine pesticides^22^, heavy metal residues^23^, and other issues, but its antibiotic residues study has not yet been carried out. Therefore, the objectives of this study were: (1) to investigate the concentration and distribution features of four quinolones antibiotics (ofloxacin (OFX), norfloxacin (NOR), ciprofloxacin (CIP), and enrofloxacin (ENR)) in water, sediment and fish from the Qingshitan reservoir; (2) to explore the correlation between the physical and chemical indicators of water, sediment and quinolones antibiotics contamination via correlation analysis; (3) to assess the risk quotients (RQ) for antibiotics in sediment; (4) to evaluate the human health risk of a selected antibiotic in the fish muscle through the average concentration measurement method. Furthermore, it can reveal the pollution status and risk level of antibiotics in water, sediments, and fishes that will provide a scientific basis for antibiotic pollution control.

## Materials and methods

### Study area

Qingshitan reservoir is located in the upstream of the Gantang river, northwest of Guilin City, Guangxi and is about 30 km^2^^20^. It is a typical large-scale mountainous reservoir in the northern Guangxi region^24^ as a special artificial lake ecosystem^25^. This complex aquatic ecological environment provides habitat for fish reproduction besides being used for irrigation, aquaculture, tourism, flood protection, as well as water-supply of the Lijiang River^26^. It’s located at subtropical monsoon climate, and the annual average temperature of the reservoir is 18.6 °C, rainfall is 2,400 mm, evaporation is 1682 mm, and runoff is 8.4 × 10^8^ m^3^^21^. In 2005, the area used for cage fish in the reservoir was 30,000 m^2^, which reached nearly 100,000 m^2^ in 2013 and as a result, the safety of drinking water in Guilin city was in danger. In order to improve the water quality of the reservoir, the government cleared up the entire cage fish culture in 2013, to completely eliminate this source of pollution but were replaced with a new breeding model—cofferdam fish culture at the same time. The feed and faeces produced by fish in this breeding mode are fed into Qingshitan reservoir, which also affects the water quality and ecology. Therefore, this study focuses on the concentration and the impacts of antibiotics in the Qingshitan reservoir.

### Sample collection

**Figure 1:**
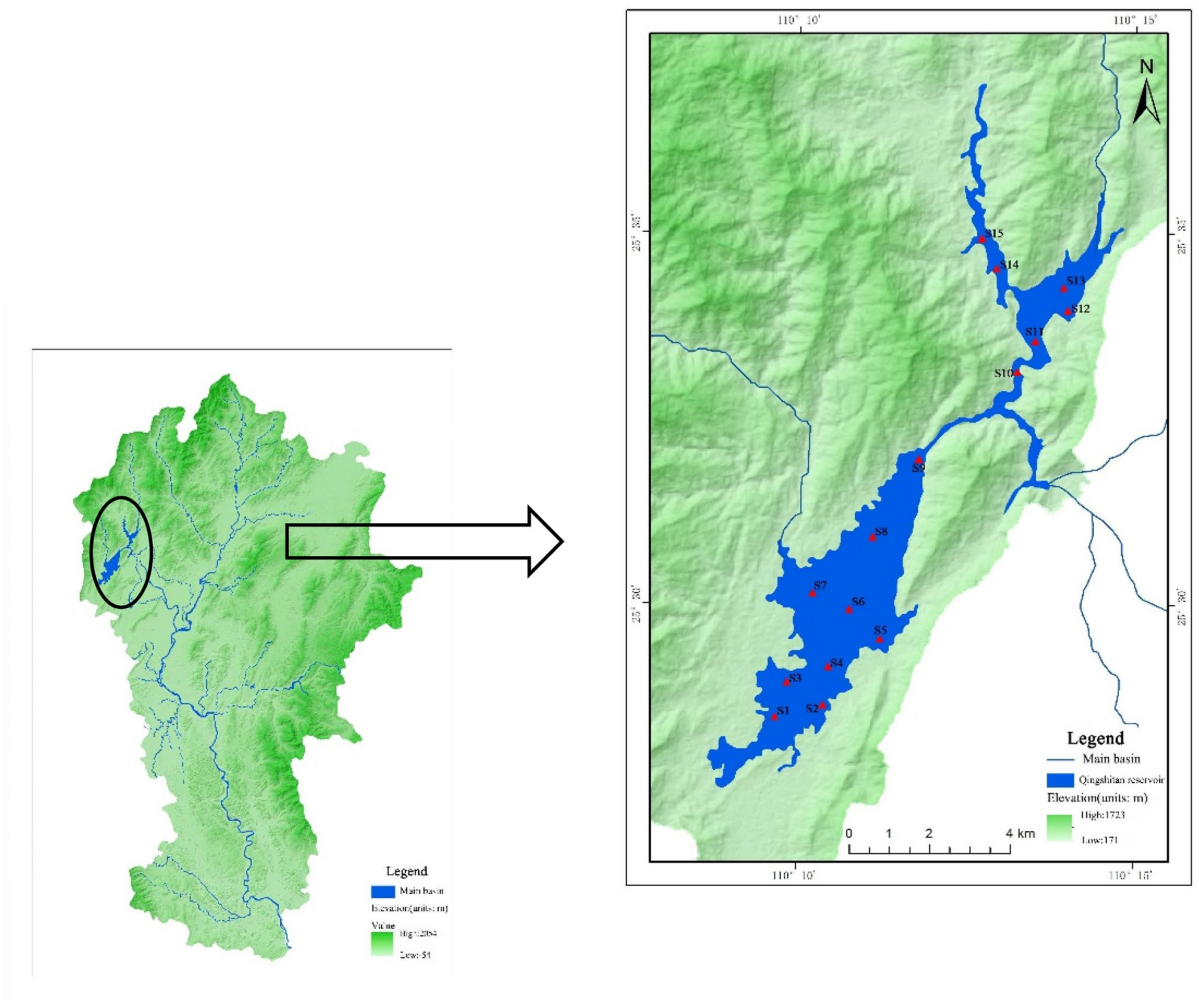
The 15 sampling points (S1–S15) of Qingshitan reservoir (Guangxi, South China), image extracted from ArcGIS 10.2.

Based on the environmental and hydrological characteristics of the Qingshitan reservoir and its main entry tributaries, 15 sampling sites (S1–S15) were deployed in this study, including 9 in the west lake and 6 in the east lake (Fig. 1). For water, sediment and fish samplings the following were carried out respectively. From 0 to 50 cm depth, 2 L surface water samples were collected by a stainless steel water container, then immediately loaded into a brown glass bottle and labeled. The samples were stored in a 4 °C refrigerator and pre-treated within 48 h. For sediments, the 0–5 cm top surface from the lake bottom was collected by a mud grab sampler (for S6 did not collect samples). Three sediment samples collected in each sampling point along the channel section for 150–200 g per sample to mix uniformly. Then the samples were placed in a sterile plastic bag quickly, transported to the laboratory, and stored at − 20 °C in the dark. As for the fish, seven species (3 pieces each) were purchased from the surrounding markets of west and east lake include: *Ctenopharyngodon idellus*, *Cyprinus carpio*, *Carassius auratus*, *Sinibrama macrops*, *Silurus asotus*, and *Pelteobagrus fulvidraco* and *Hemiculter leucisculus*, and were transferred to the laboratory to process within 24 h. Fish muscles and skin were prepared, homogenized and stored at − 20 °C before the analysis.

### Sample preparation and detection

#### Sample extraction

Antibiotics were extracted from the water samples according to the method reported in the literature^27,28^, extracted from sediments following the reported method by Zhou et al.^29^ and Liang et al.^22^, and extracted from fishes following the reported method by He et al.^30^. Conductivity, dissolved oxygen (DO), pH, temperature and turbidity of each sample were measured by portable water quality analyzer (HACH-HQ40D) and turbidity meter (HACH-2100Q). The contents of TOC, TN, and TP in water samples were analyzed following standard methods^31^.

#### Sample analysis

All samples (water, sediment, and fish) were analyzed via high-performance liquid chromatography (HPLC). A 20 μL of the processed sample was injected into a Waters C18 column (4.6 × 150 mm, 5 μm) at 25 °C. The flow rate was 1 mL/min and the eluent was composed of acetonitrile (A) and 0.0067 mol/L phosphoric acid solution (B). 15% mobile phase A was maintained in this program during the whole process. The fluorescence detector was used in this analysis with emission wavelength of 450 nm, and excitation wavelength of 280 nm.

### Quality control and quality assurance

The antibiotic concentration in all samples was quantified by an external standard method. Related studies have shown that the sample matrix has a certain influence on the determination of the standard curve^32^. A series of samples with spiked concentrations from 0.02 to 2 μg/mL and 10 to 1,000 μg/kg of individual antibiotics were used to calculate the calibration curves (R^2^ > 0.99). Each batch of samples was blanked and the sample spiked at the same time as the preparation for quality control. Three parallel samples were prepared for each spiked concentration and the results of the recovery were expressed as the average of three replicates. The spiked concentrations of water samples were 0.05 μg/mL and 0.2 μg/mL and the recoveries ranged from 72.06% to 90.74%. The spiked concentrations of the sediment samples were 50 μg/kg and 500 μg/kg while the recoveries ranged from 61.32% to 92.39%. The spiked concentrations of fish samples were 10, 100 and 1,000 μg/kg and the recoveries of the four antibiotics ranged from 73.35% to 99.52%. The standard deviation was less than 20%.

### Ecological risk assessment in water

The antibiotics in the environment are new organic pollutants, and their concentration in the environment is lower than that of conventional pollutants, generally in order of magnitude ng/L ~ μg/L. The risk quotient (RQ) method was used to evaluate the potential risk of antibiotic residues on aquatic ecosystems. The formula used was as follow^33,34^:

$${\text{RQwater}} = {\text{MEC}}/{\text{ PNECwater}}$$

$${\text{PNECwater}} = {\text{LC}}_{50} {\text{EC}}_{50} {\text{/AF}}\;{\text{or }}\;{\text{PNECwater}} = {\text{NOEC/AF}}$$

where MEC is the actual measured concentration in the environment (ng/L), PNEC_water_ is the predicted no-effect concentration in the water and it is the maximum drug concentration that will not have an adverse effect on the microorganisms or the ecosystem in the environment under the existing cognition (ng/L). LC_50_ is the half lethal concentration, and EC_50_ is the half-maximum effect concentration (ng/L), LC_50_ and EC_50_ are all obtained from the literature^35,36^, and when there are multiple values, the minimum value was taken. AF is the evaluation factor, which is the recommended value of the European Water Framework Directive (1,000). When RQ ranges from 0.01 to 0.1, the ecological risk assessment of antibiotics in sediments is low. The environment is in medium risk when RQ ranges from 0.1 to 1, while RQ is more than or equal to 1, the risk is at a high level^37^.

### Ecological risk assessment in sediment

At present, the ecological risk study of antibiotics is mainly focused on water, while for the antibiotics in sediments is rarely involved^6^. In order to study the effects of quinolone antibiotic residues on the aquatic ecosystem in surface sediments of Qingshitan reservoir, risk quotient (RQ) was used to evaluate the potential risk. The formula used was as follow^33,34^:

$${\text{RQ}}_{{{\text{sediment}}}} = {\text{MEC}}/{\text{PNEC}}_{{{\text{sediment}}}}$$

$${\text{PNEC}}_{{{\text{water}}}} = {\text{LC}}_{50} {\text{/AF}}\;{\text{or}} \;{\text{EC}}_{50} {\text{/AF}}$$

$${\text{PNEC}}_{{{\text{sediment}}}} = {\text{PNEC}}_{{{\text{water}}}} \times {\text{K}}_{d}$$

$${\text{K}}_{d} = {\text{K}}_{{{\text{oc}}}} \times {\text{F}}_{{{\text{oc}}}}$$

$${\text{lgK}}_{{{\text{oc}}}} = 0.623{\text{lgK}}_{{{\text{ow}}}} + 0.873$$

where PNEC_sediment_ is the predicted no-effect concentration in the sediment (ng/L). K_d_ is the sediment–water partition coefficient, K_oc_ is the organic carbon–water partition coefficient (L/kg)^38^, F_oc_ is the content of organic carbon in the sediment (F_oc_ = 0.03 g/g), K_ow_ is the octanol–water partition coefficient. When RQ ranges from 0.01 to 0.1, the ecological risk assessment of antibiotics in sediments is low. The environment is in medium risk when RQ ranges from 0.1 to 1, while RQ is more than or equal to 1, the risk is at a high level^37^.

### Health risk assessment in water

In this study, the following formulas^15^ were used to calculate the health risk assessment of antibiotics in water.

$${\text{R}}_{{{\text{QH}}}} = \frac{{{\text{D}}_{{{\text{osea}}}} { } \times {\text{EF}} \times {\text{ED}}}}{{{\text{ADI}} \times {\text{BW}} \times {\text{AT}}}}/70$$

$${\text{D}}_{{{\text{osea}}}} = {\text{C}}_{{\text{i}}} \times {\text{k}}_{{\text{T}}} \times {\text{IR}}_{{{\text{DW}}}}$$

where D_osea_ is the exposure dose (μg/(person d)). EF is the exposure frequency of target pollutants (day/year). ED is the exposure time (a). ADI is the allowable daily intake (μg/(kg·d). BW is the adult weight (kg). AT is the average human exposure to drugs (d). C_i_ is measured concentration of target pollutants (μg/L). k_T_ is the remaining proportion of antibiotics in water after the water treatment process. IR_DW_ is the daily average water intake (L/d)^39^.

This study uses adult weight as a reference, and the parameters refer to the values recommended by the US Environmental Protection Agency as follows: EF is 350 day/year, ED is 2 years. BW is 70 kg. AT is 10,950. K_t_ is 1. IR_DW_ is 2 (https://www.epa.gov/risk/guidance-selecting-age-groups monitoring and assessing-childhood).

### Data analysis

Statistical analyses and correlation analyses were performed using IBM SPSS 19.0. The Pearson correlation was used to analyze the relationship (1) between the deposition of physical and chemical indicators of water, sediments, and antibiotic concentrations, and (2) between antibiotic concentrations in different feeding habits and habitant layers. The antibiotic concentrations between different sampling points also have been discussed.

## Results

### Concentration of antibiotics and basic quality parameters in water

**Figure 2:**
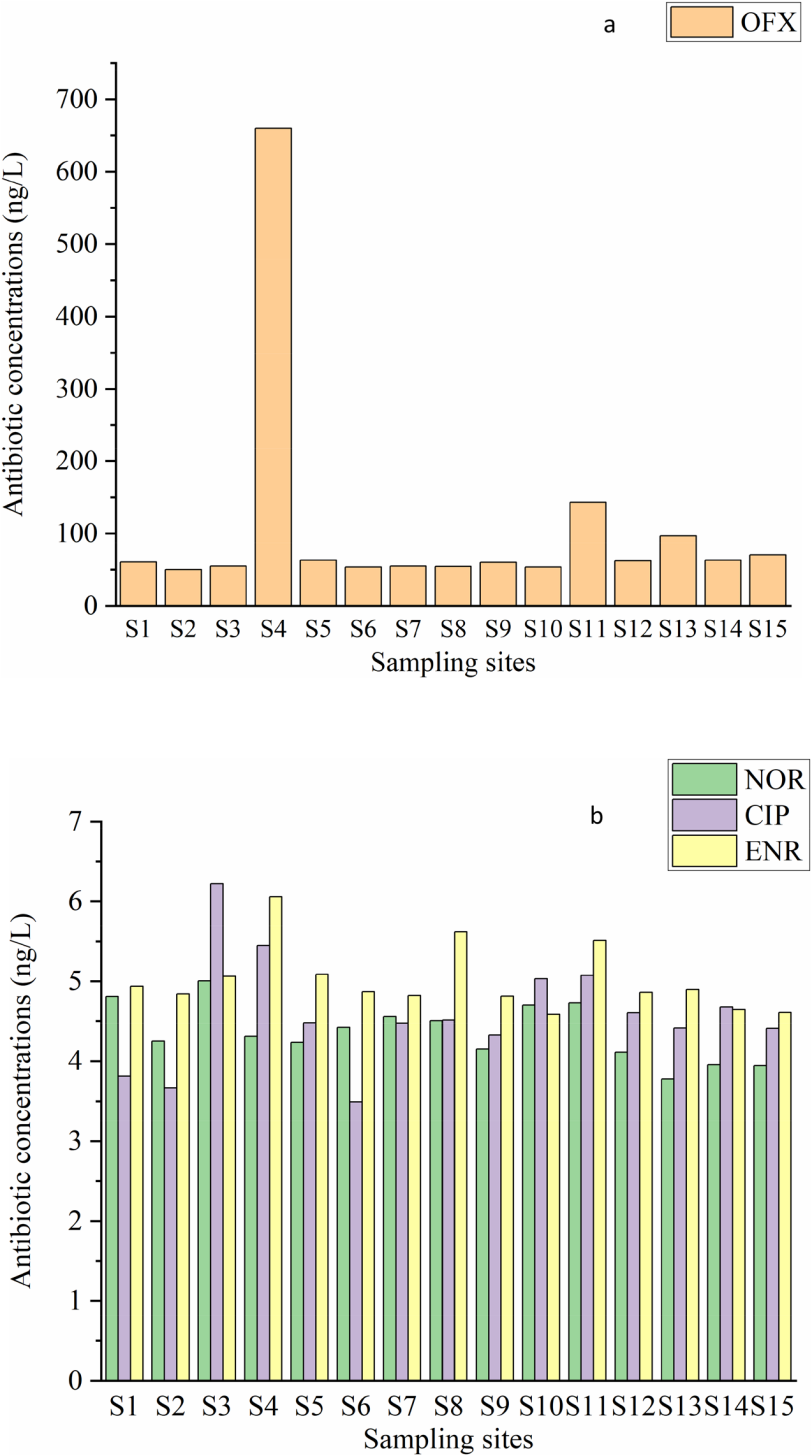
Quinolone antibiotic (OFX) (**a**), and (NOR, CIP, ENR) (**b**) concentrations of water samples in ng/L at each sampling point of Qingshitan reservoir.

The antibiotics concentration of water (Fig. 2) is ranged from 3.49 to 660.13 ng/L. The rank of four antibiotics was as follows NOR < CIP < ENR < OFX. The highest antibiotic concentration was observed at sampling point S4 and the lowest at S2. The mean concentration of OFX, NOR and ENR in the west lake were higher than that in the east, while CIP was close to the east lake, indicating that the antibiotics pollution of water in the west lake was more serious than that in east lake.

**Figure 3:**
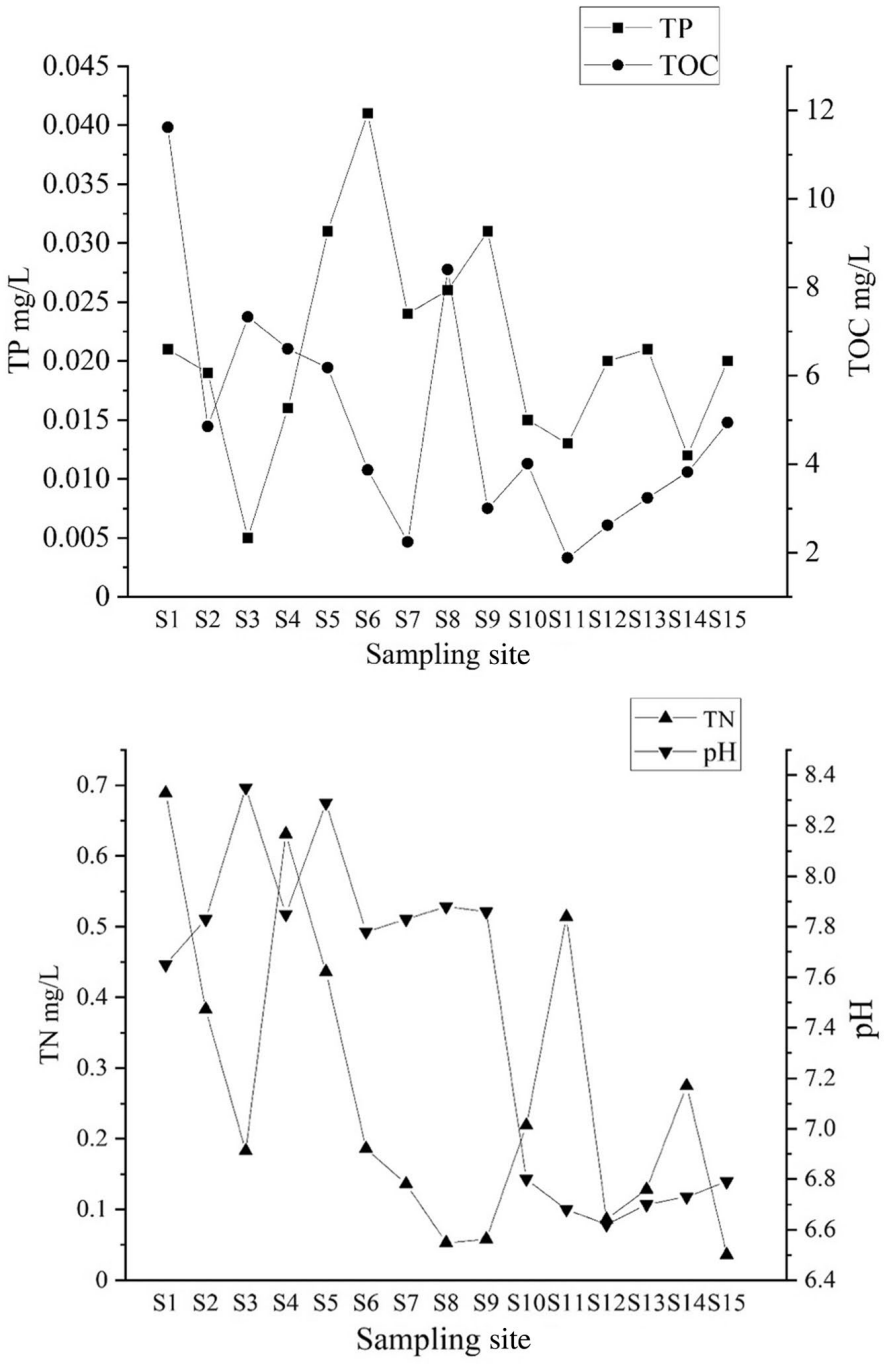

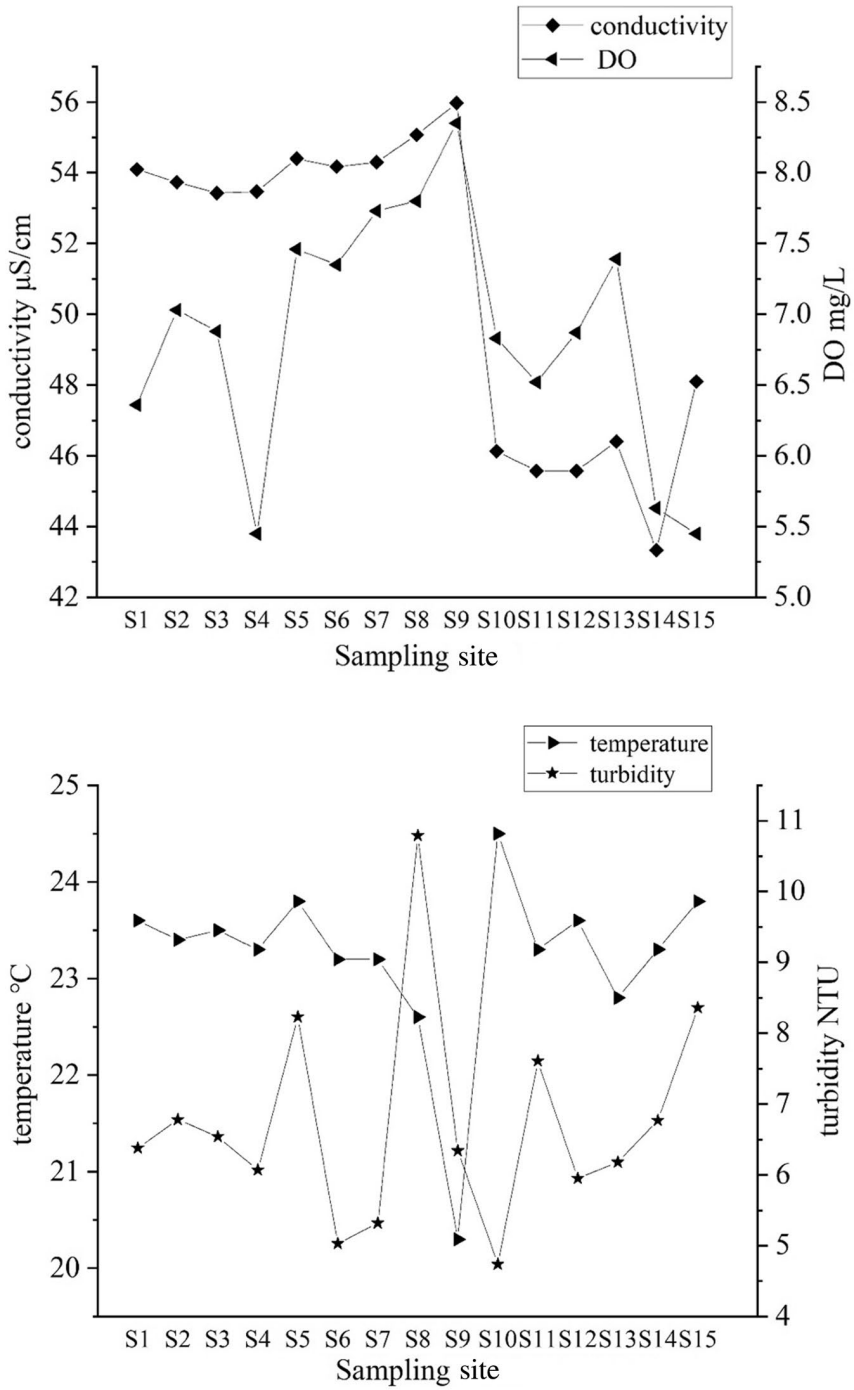
Basic parameters of water (TP, TOC, TN, pH, conductivity, DO, temperature, and turbidity) at each sampling site of Qingshitan reservoir.

The result showed that TP ranged from 0.005 to 0.041 mg/L and the maximum was observed at S6. The range of TOC was from 1.88 to 11.62 mg/L, while TN was from 0.036 to 0.689 mg/L. The maximum TOC and TN were observed at S1, while the minimum was at S11 and S15, respectively. The pH and conductivity in the west lake were all higher than that in the east lake. The range of DO was from 5.45 to 8.35 mg/L and the water temperature was 20.3–4.5 °C, while turbidity was from 5.03 to 10.79 NTU (Fig. 3).

### Correlation between basic quality parameters and quinolone antibiotics in water

The concentration of NOR showed a significant relationship with pH (R^2^ = 0.401, *P* < 0.01), while a positive relationship could be found between NOR and turbidity (R^2^ = 0.352, *P* < 0.05). There was a negative relationship between CIP and TP (R^2^ =  − 0.345, *P* < 0.05), while no relationship between basic parameters and OFX and ENR in water was found. The results indicated that pH and turbidity may be important factors affecting the distribution of NOR, while TP concentration is an important factor affecting the distribution of CIP concentration (Table 1).

**Table 1:** Correlations between four Quinolone antibiotics concentrations and eight water quality parameters from Qingshitan Reservoir.

	OFX	NOR	CIP	ENR
TP	− 0.071	0.053	− 0.345*	0.111
TOC	− 0.097	0.033	0.028	0.146
TN	0.146	0.195	− 0.052	0.29
pH	− 0.2	0.401**	− 0.03	0.187
Turbidity	− 0.12	0.352*	− 0.216	0.253
DO	− 0.226	0.071	− 0.142	0.109
Temperature	− 0.12	− 0.206	0.028	− 0.094
Conductivity	0.06	0.181	0.233	0.087

*Significantly related at the 0.05 level.

**Significantly related at the 0.01 level.

### Concentration of antibiotics and basic parameters in sediment

**Figure 4:**
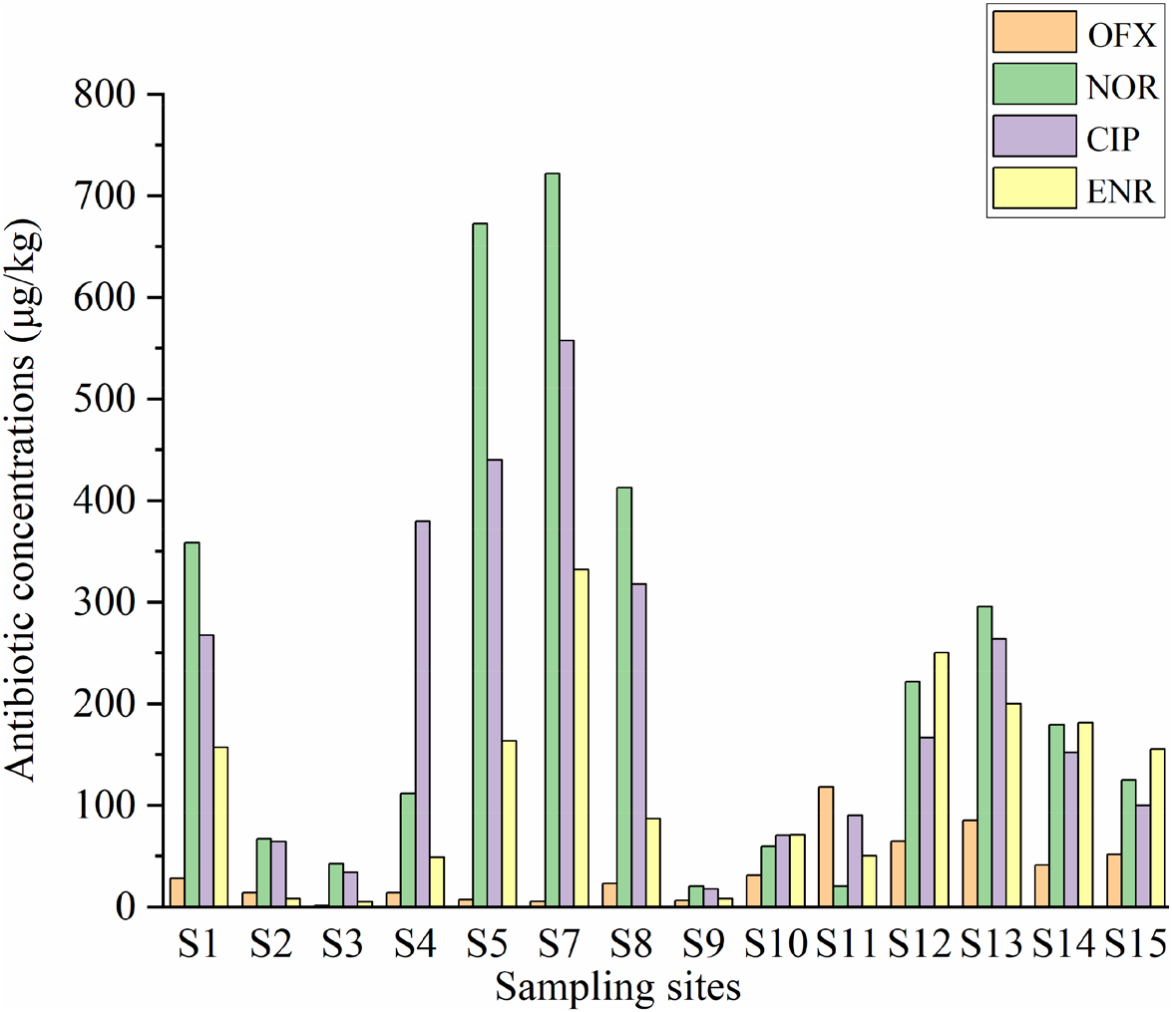
Quinolone antibiotic (OFX, NOR, CIP, and ENR) concentrations of sediments in μg/kg at each sampling point of Qingshitan reservoir.

The antibiotic concentration of sediments is shown in Fig. 4. All four types of antibiotics were detected in the sediments at each sampling point. The concentration ranged from 1.03 to 722.18 μg/kg. The rank of four antibiotics was as follows OFX < ENR < CIP < NOR. The highest quinolone antibiotic concentration was observed at sampling point S7 and the lowest at S9. The highest concentration of NOR and CIP were found at S7 and the lowest at S9. Moreover, the greatest concentrations of ENR and OFX recorded at S7 and S1, respectively, while the lowest at S3 sampling point. The mean concentration of antibiotics in the west lake (1.03 ~ 722.18 μg/kg) was higher than that in the east (20.17 ~ 295.14 μg/kg).

**Figure 5:**
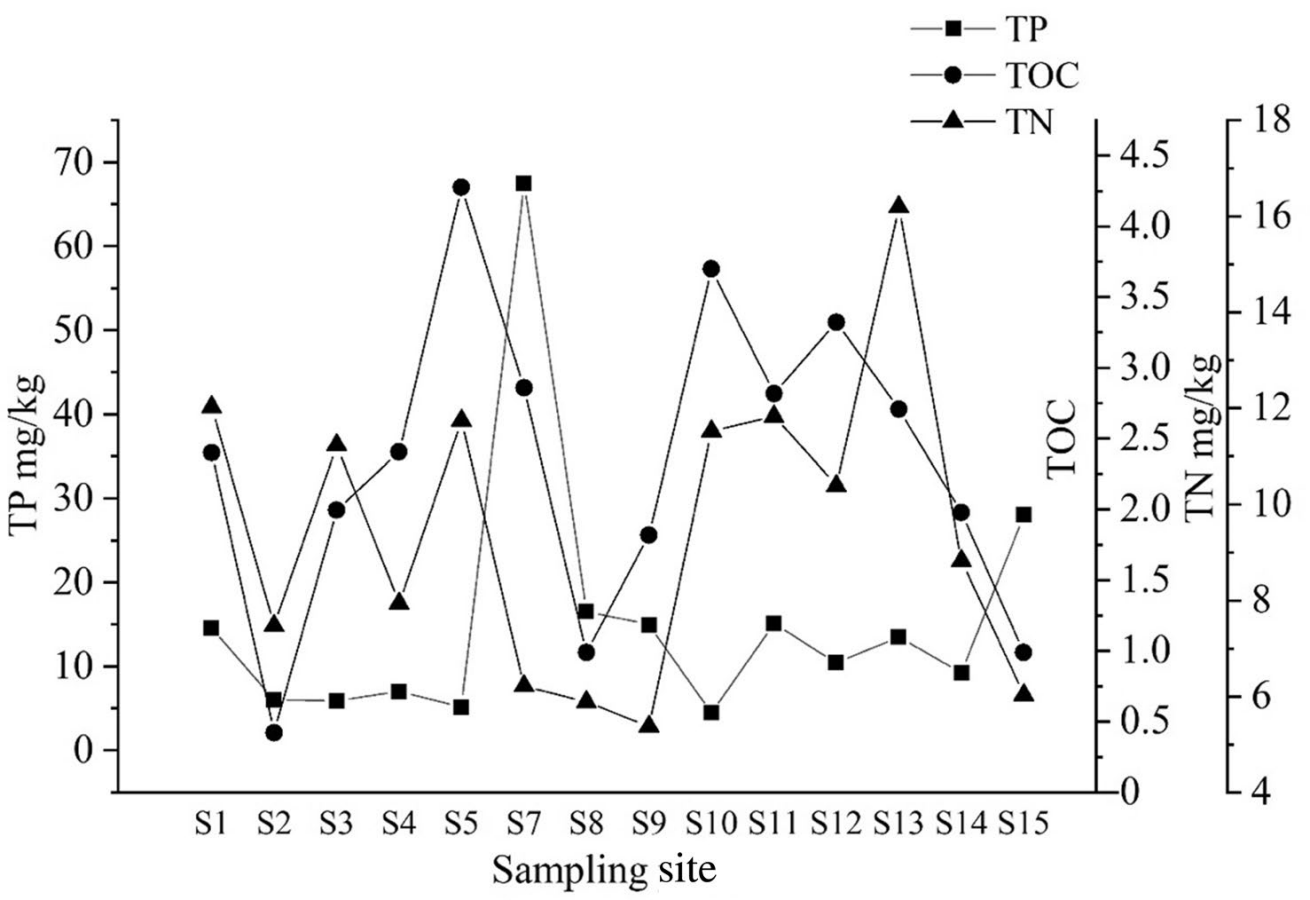
Basic parameters of sediments (TN, TOC, and TP) at each sampling site of Qingshitan reservoir in mg/kg.

The results showed that TOC ranged from 0.42% to 4.28% with an average of 2.33% while TOC at S5 was the maximum. The range of TP was from 4.49 to 67.45 mg/kg with an average of 15.61 mg/kg and the maximum value was observed at S7 point. TN ranged from 5.38 to 16.20 mg/kg and the average value was 9.48 mg/kg with the maximum measured value at S13 (Fig. 5).

### Correlation between basic parameters and quinolone antibiotics in sediment

The sediment samples were performed to explore the correlations between the basic parameters and antibiotic concentrations (Table 2). The levels of NOR showed significant relationships with TP (R^2^ = 0.372, *P* < 0.05) and TOC (R^2^ = 0.308, *P* < 0.05). In addition, a positive relationship could be found between the concentrations of OFX and TN (R^2^ = 0.357, *P* < 0.05). The levels of ENR presented significantly positive relationships with TP (R^2^ = 0.336, *P* < 0.05). However, no significant relationships between CIP and all basic parameters of sediment were observed.

**Table 2:** Correlation between three basic parameters of sediments and concentrations four of quinolone antibiotics.

Analytes	TP	TOC	TN
OFX	− 0.068	0.159	0.357*
NOR	0.372*	0.308*	0.014
CIP	0.284	0.269	− 0.002
ENR	0.336*	0.221	0.002

*Significantly related at the 0.05 level.

### Antibiotics in fish

The concentration of quinolone antibiotics in fish are shown in Table 3. The antibiotic contents in fish were orderly as NOR < ENR < CIP < OFX, ranging from 6.73 to 968.66 μg/kg. Fish with the highest OFX and NOR concentration were *C.idellus* and *C.auratus*, while for CIP and ENR were *S.macrops* and *H.leacisculus*, respectively.

**Table 3:** Quinolone antibiotic concentrations of seven types of fish in Qingshitan reservoir (μg/kg).

Fish	OFX	NOR	CIP	ENR
*Carassius auratus*	148.91	33.27	27.56	44.58
*Cyprinus carpio*	131.7	25.28	21.63	14.21
*Pelteobagrus fulvidraco*	372.3	21.7	12.15	6.73
*Sinibrama macrops*	58.59	28.51	80.26	8.87
*Hemiculter leucisculus*	91.74	25.41	73.53	102.87
*Ctenopharyngodon idellus*	370.83	9.15	13.67	47.43
*Silurus asotus*	968.66	12.12	16.61	17.92

*OFX* ofloxacin, *NOR* norfloxacin, *CIP* ciprofloxacin, *ENR* enrofloxacin.

**Figure 6:**
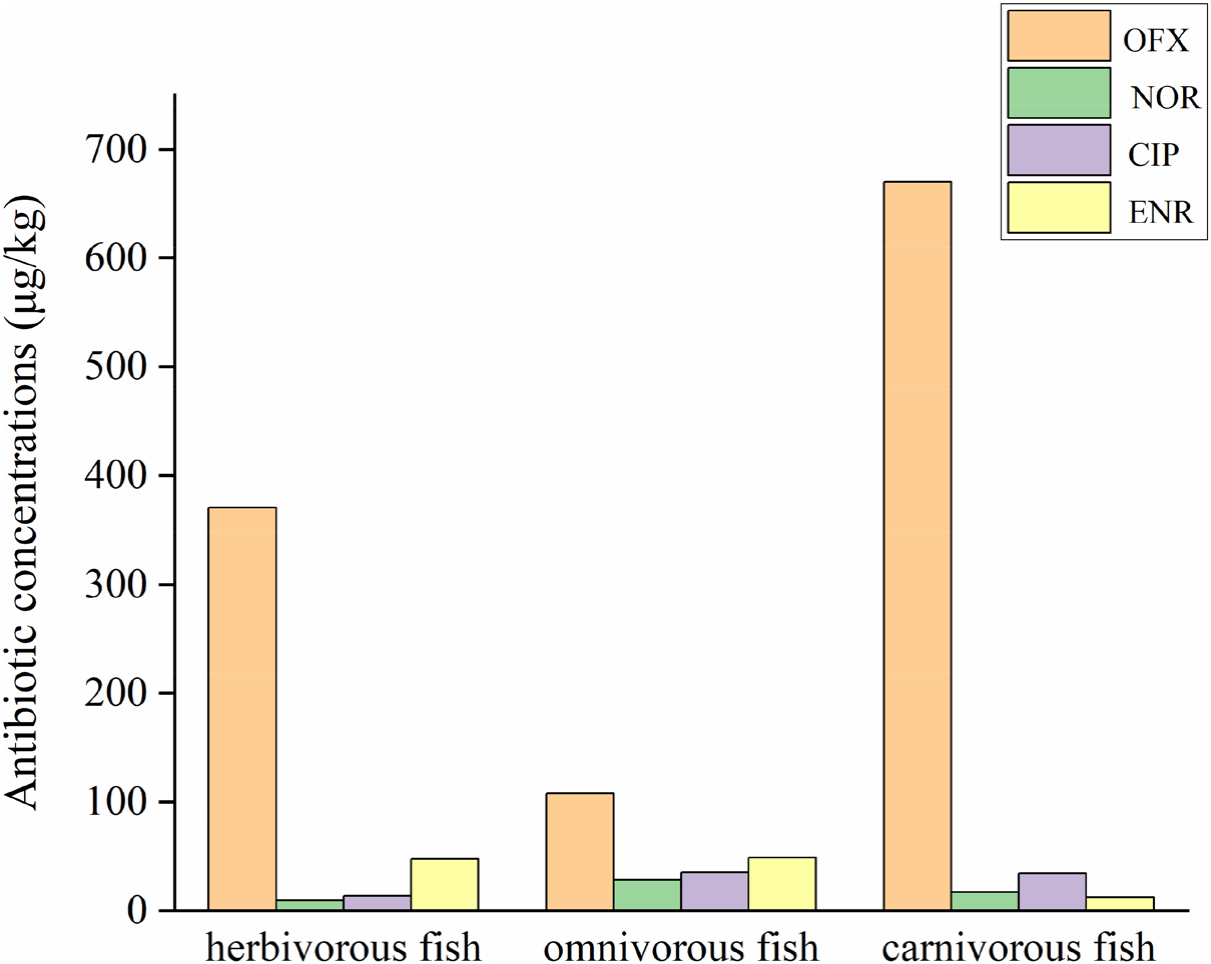
Antibiotic concentrations (OFX, NOR, CIP, and ENR) of different fish feeding habits of Qingshitan reservoir in μg/kg. *Significantly related at the 0.05 level.

The quinolones residues in fish are related to species, feeding habits, and metabolic functions^40^. The feeding habits of collected fish samples are omnivorous (*Cyprinus carpio*, *Carassius auratus*, *Sinibrama macrops*, *Hemiculter leucisculus*), herbivorous (*Ctenopharyngodon idellus*), and carnivorous (*Silurus asotus*, *Pelteobagrus fulvidraco*). The results of antibiotic residues in different fish feeding habits showed that the highest concentration of OFX found in carnivorous fish and the lowest was omnivorous fish while the highest and lowest concentration of NOR were omnivorous and herbivorous fishes, respectively. The highest content of CIP and ENR were detected in omnivorous fish and the lowest content was detected in herbivorous and carnivorous fishes, separately (Fig. 6). The relationship between omnivorous fish and carnivorous fish of OFX concentration was significant (*P* < 0.05). There was a significant relationship between omnivorous fish and herbivorous fish of NOR concentration (*P* < 0.05). The relationship between omnivorous, carnivorous, and herbivorous fishes CIP concentration was significant (*P* < 0.05). There was no relationship between ENR concentration in all kinds of fish.

**Figure 7:**
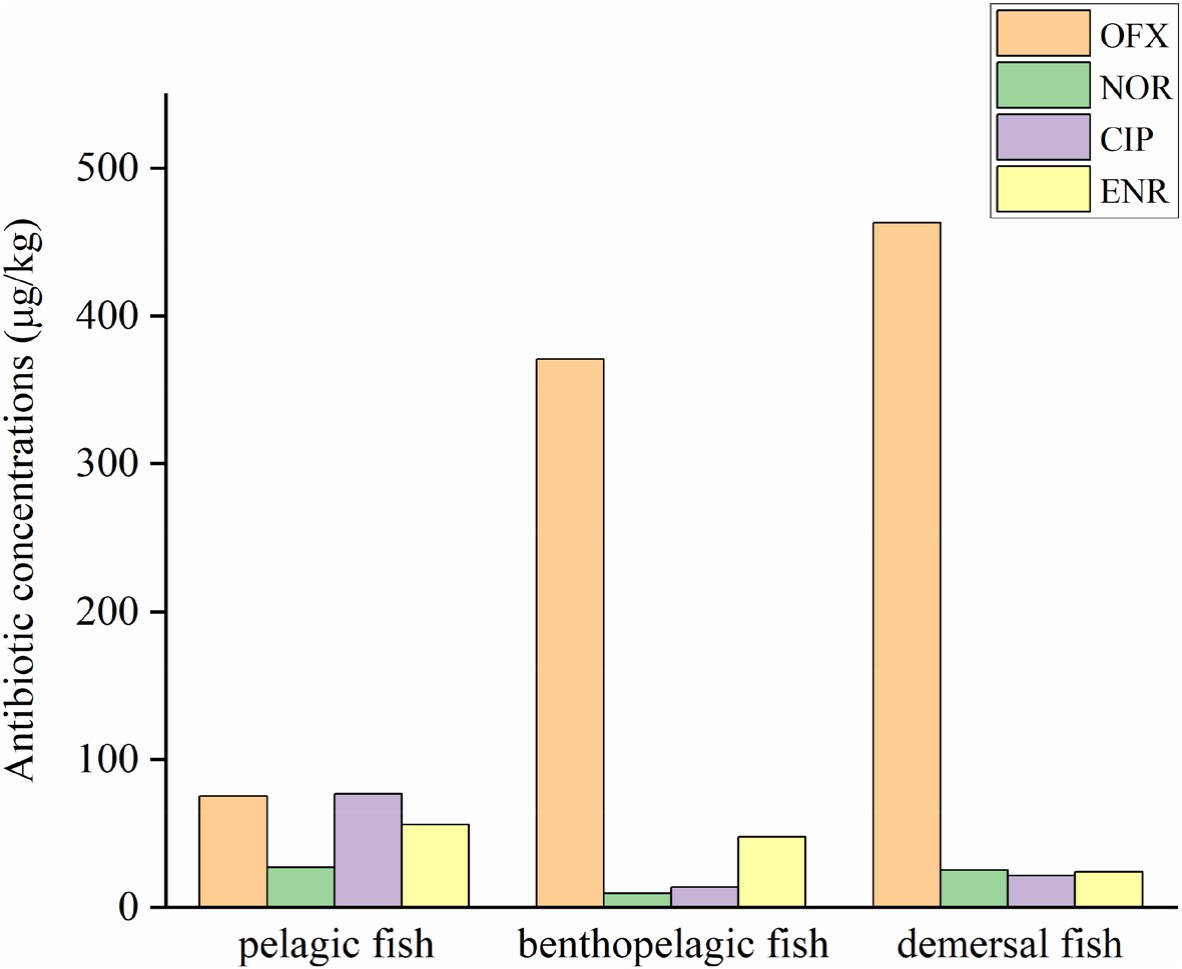
Antibiotic concentrations (OFX, NOR, CIP, and ENR) of fish in different habitat layers of Qingshitan reservoir in μg/kg. *Significantly related at the 0.05 level. **Significantly related at the 0.01 level.

The habitat aquifers of collected fish samples are pelagic (*Sinibrama macrops*, *Hemiculter leucisculus*), benthopelagic (*Ctenopharyngodon idellus*), and demersal (*Cyprinus carpio*, *Carassius auratus*, *Silurus asotus*, *Pelteobagrus fulvidraco*)^41^. The results show that the highest concentration of OFX was found in demersal fish, and the lowest was found in the pelagic fish. The concentrations of NOR, CIP, and ENR were highest in pelagic fish and lowest in benthopelagic, benthopelagic, and demersal fishes, respectively (Fig. 7). The relationship between pelagic and demersal fish of OFX concentration was extremely significant (*P* < 0.01). There was a significant relationship between pelagic and benthopelagic fish in NOR concentration (*P* < 0.05). The relationship between benthopelagic and demersal fish of NOR concentration was extremely significant (*P* < 0.01). There was no relationship between CIP and ENR concentration in each aqua layer of fish.

### Risk assessment in water, sediment, and fish

The results of antibiotics ecological risk assessment in water (Table 7), sediment (Table 8), as well as health risk assessment of edible fish (Table 9) of Qingshitan reservoir are tabulated. Outcomes revealed that the highest RQ values in water was for OFX (RQ > 1) antibiotic only, while for sediment all four types of antibiotics were having RQ values of greater than 1. As for the health risk, in order to comparatively analyze the obtained values, maximal acceptable and neglectable risk levels of water by foreign organizations, and estimated daily intake (EDI) are shown in Tables 10 and 11, respectively. The ENR values of fish muscle in Qingshitan reservoir were mainly below the maximum acceptable level.

## Discussion

### Residual characteristics of quinolone antibiotics in water, sediment, and fish

Antibiotics in the environment mainly come from pharmaceutical, medical, aquaculture, and domestic wastewater^28,42–44^. Quinolone antibiotics are considered to be ideal antibacterial drugs for humans and animals, they have been widely used in livestock and poultry breeding^45^ for the treatment of bacterial diseases due to their broad spectrum of antibacterial activity, strong antibacterial activity, no cross-resistance to other antibiotics, and low toxic side effects, etc.^35^.

Compared with the concentration of antibiotics in rivers and lakes worldwide, the concentration of the antibiotic in the Qingshitan reservoir water was at a moderately low level (Table 4). Of the four quinolone antibiotics, the highest concentration of antibiotics in water was OFX, and there were no significant differences among the values for the other three types at the entire sampling points. This result was similar to some other researches^46–48^. The highest water OFX was observed at S4 point which is located near the Goping countryside, with a high population density. OFX is an antibacterial drug shared by humans and animals, while the manure of humans and urban sewage is often used as an organic fertilizer for planting crops^41^. Besides, the concentration of the antibiotics at the lakeside was averagely higher than that at the center of the lake. This is because the lakeside water is shallower, tends to stagnate, and it is more susceptible to be polluted by land sources^49^.

**Table 4:** Concentrations of quinolone antibiotics (OFX, NOR, CIP, and ENR) in different areas.

Study area	OFX/(ng/L)	NOR/(ng/L)	CIP/(ng/L)	ENR/(ng/L)	References
Hunhe river, China	nd ~ 280	nd ~ 1,380	nd ~ 65	nd ~ 17	^66^
Bohai sea, China	3 ~ 5,100	32 ~ 6,800	4.9 ~ 390	–	^61^
Haihe river, China	180	–	130	–	^29^
Baiyangdian lake, China	0.38 ~ 32.6	nd ~ 156	nd ~ 60.3	nd ~ 4.42	^74^
Laizhou bay, China	nd ~ 45.5	nd ~ 572	nd ~ 346	nd ~ 24.6	^72^
Xiaoqing river, China	9.5 ~ 1605	nd	nd ~ 56.6	nd	^47^
Huangpu river, China	nd ~ 6.5	nd ~ 2.6	nd ~ 2.7	nd	^46^
Tai lake, China	14 ~ 474	59 ~ 271	18 ~ 269	19 ~ 229	^48^
Pearl river, China	7.1	67.5	–	nd	^28^
Victoria harbor, China	660 ~ 6,840	14 ~ 2,290	–	–	^75^
Lambro river, Italy	306	–	26,200	–	^76^
Surface water in southeast Queensland, Australia	–	1,150	300	1,300	^77^
Surface water in East Aurora, USAhttps://www.baidu.com/javascript	–	–	nd ~ 360	–	^78^
Qingshitan reservoir	50.0 ~ 660.13	3.70 ~ 5.00	3.49 ~ 6.22	4.59 ~ 6.06	This research

Nd indicates that it has not detected the antibiotic, – indicates that the antibiotic has not been tested.

On the other hand, sediments play an important role in the degradation and adsorption of pollutants in rivers and lakes^50^. Compared with the concentration of antibiotics in sediment worldwide, in the Qingshitan reservoir, the values recorded were higher than those of other regions (Table 5). Among the four quinolone antibiotics, the concentration of NOR in sediment was the highest, which might be due to being widely used as a feed additive in aquaculture and animal husbandry. The livestock and poultry husbandry wastewater is directly discharged into the reservoir area and the NOR in the wastewater is adsorbed in sediments. Basically, NOR is easier to be adsorbed in sediment than other three antibiotics^51^. When NOR first enters the sediment environment, it has a certain inhibitory effect on microorganisms, while microbial has little impact on the degradation of norfloxacin^52,53^. Besides, the degradation rate of NOR is slower than the other three antibiotics^54,55^.

**Table 5:** Quinolone antibiotic concentrations (OFX, NOR, CIP, and ENR) of sediments in different areas (mean/range of contents, μg/kg).

Study area	OFX	NOR	CIP	ENR	References
Jiaozhou Bay, China	nd ~ 33.83	–	nd ~ 11.52	nd ~ 1.92	^68^
Yellow River, China	3.07	8.34	32.8	nd	^29^
Haihe River, China	10.3	32	16	nd	
Liaohe River, China	3.56	3.32	nd	nd	
Qinghe River, China	5.9	49.1	28.3	2.2	^50^
Pearl River Estuary, China	nd ~ 2.08	50.24 ~ 153.06	–	nd ~ 25.62	^28^
Griffin Lake, Switzerland	–	2.4	2.52	–	^79^
Northwest River, New Jersey, USA	< 21	–	< 10	–	^80^
Qingshitan reservoir	1.03 ~ 118.11	20.17 ~ 722.18	17.48 ~ 557.18	4.70 ~ 331.82	This Research

Nd indicates that it has not detected the antibiotic; – indicates that the antibiotic has not been tested.

In general, the content of quinolone antibiotics in the sediments of the west lake was higher than that in the east lake, probably due to the following reasons. First, the western side of the west lake is adjacent to the Gongping township, and the area is larger than the east lake. In contrast, the area around the east lake is steep, the lake is narrow, and there are fewer pollution sources around it^49^. As it is shown in Fig. 1, the reservoir is in fact including two lakes connected through a relatively long waterway. These two are naturally separated due to topographical characteristics of the watershed and hence geographically distinguishable. Second, the number of surrounding aquaculture farms and livestock farms in the west is also greater than those of the east lake; while, the farmlands are widely distributed along the shore of the west lake that potentially causing more pollution resources. Since Qingshitan reservoir watershed is rich in rainfall^56^ throughout the year, hence the lands around the reservoir area are being washed by rain, resulting in the antibiotics carried by runoff. Also, the excrement enters the reservoir area by rainwater and then becomes sediments, thereby increases the antibiotics content in sediments as well as water. At the same time, due to the quinolone's strong adsorption capacity on particulate matter and sediment^37^, the irrigated croplands water also contains high concentrations of antibiotics that discharge into the reservoir area without adequate treatment.

Compared with other domestic results, OFX in the fish muscles of Qingshitan Reservoir was higher, while the contents of NOR, CIP and ENR were similar to other regions (Table 6). In general, this can be related to the type of drug used, the amount of drug used, the exposure time to drugs, and the type of fish in different areas. The highest concentration of antibiotics in fish was OFX and the lowest was NOR, which was related to the large usage of OFX. This is similar to the concentration of quinolone antibiotics in water but contrary to the sediments. The fish species with the highest concentration of antibiotics was *S.asotus.* It may be related to the level of contamination of different regions and the growth environment of fish. It may also be relevant to the living habits, the food intake in the growth stage and the food chain grade of different fish species^57^. *S.asotus* is benthopelagic fish and inhabits in slow flow and static water, where the renewal cycle is so slow that antibiotics are easy to accumulate. In addition, studies^58^ have shown that quinolones can be absorbed quickly, eliminated slowly and enriched in fish. Thus, resulting in a high content of quinolones in the muscle of *S.asotus.* The *S.asotus* are usually at a high nutritional level in the food chain. Harmful substances accumulate from the producer and will eventually enter *S.asotus* along with the food chain. This accumulation will increase as time passes.

**Table 6:** Quinolone antibiotics concentrations (OFX, NOR, CIP, and ENR) of fish muscles in different regions (μg/kg).

Study area	Fish species	OFX	NOR	CIP	ENR	References
Guangdong,China	*Silver carp*	–	103.03	27.07	34.20	^40^
	*Cyprinus carpio*	–	46.64	53.50	18.20	
	*Hypophthalmichthys nobilis*	–	67.97	101.09	2.20	
	*Parabramis pekinensis*	–	98.73	165.15	2.30	
	*Acipenser sturio*	–	106.85	94.64	1.00	
Guiyang,China	*Pelteobagrus fulvidraco*	nd	nd	nd	0.30	^57^
	*Leiocassis longirostris*	29.30	nd	nd	72.90	
	*Lateolabrax japonicus*	385.73	nd	16.37	312.00	
	*Cyprinus carpio*	98.7	nd	nd	75.77	
Hongze lake,China	*Silver carp*	–	–	24	–	^81^
	*Cyprinus carpio*	–	–	16	–	
	*Mylopharyngodon piceus*	–	–	15	–	
	*Siniperca chuatsi*	–	–	–	–	
Jining,China	*Carassius auratus*	–	–	2.08	nd	^82^
	*Cyprinus carpio*	–	–	10.03	1.48	
	*Ophiocephalus argus Cantor*	–	–	33.8	3.25	
Suzhou, China	*Carassius auratus*	nd	–	nd	12.7	^72^
	*Lateolabrax japonicus*	nd	–	nd	34.3–64.3	
	*Parabramis pekinensis*	nd	–	nd	53	
	*Pelteobagrus fulvidraco*	4.35	–	nd	3.7–90.6	
	*Scophthalmus maximus*	nd	–	nd	16.3	
	*Siniperca chuatsi*	nd	–	33.7	9.65	
	*Trichiurus lepturus*	nd	–	nd	12.3	
Qingshitan reservoir	*Carassius auratus*	148.91	33.27	27.56	44.58	This Research
	*Cyprinus carpio*	131.7	25.28	21.63	14.21	
	*Pelteobagrus fulvidraco*	372.3	21.7	12.15	6.73	
	*Sinibrama macrops*	58.59	28.51	80.26	8.87	
	*Hemiculter leucisculus*	91.74	25.41	73.53	102.87	
	*Ctenopharyngodon idellus*	370.83	9.15	13.67	47.43	
	*Silurus asotus*	968.66	12.12	16.61	17.92	

Nd indicates that it has not detected the antibiotic; – indicates that the antibiotic has not been tested.

The concentration of antibiotics in fish is related to its feeding and living habitat. The antibiotic concentration of carnivorous fish is the highest because carnivorous fish is at the highest trophic level in the food chain. Contaminants accumulate along with the food chain, and the higher the consumer level, the higher concentration of pollutants. The concentration of antibiotics in demersal fish is higher than that in benthopelagic fish, which may be related to the photodegradation of quinolones. The results of Zhang et al*.* showed that quinolone antibiotics are almost completely free of hydrolysis in the absence of light^59^. As the depth of water increases, the light intensity becomes weaker due to turbidity. The quinolones in upper water are easier to decompose than the lower ones, so the pelagic and benthopelagic fish are less contaminated by antibiotics. Quinolone antibiotics have a strong adsorption capacity and can easily adsorb in particulates and sediments^40,60^. Antibiotics from the bottom sediment can be released into the water, and as a result, the concentrations of quinolone antibiotics in bottom water were higher than those in the upper layer. Therefore, the antibiotic content of the demersal fish was higher than that of the pelagic fish.

### Relationship between quality parameters and antibiotics in water & sediment

When the antibiotic enters the water, biotransformation processes such as adsorption and degradation will occur, on which the basic parameters such as pH, TP and TN may have important effects^61^. In this study, the CIP concentration had a significant negative correlation with the TP concentration. However, NOR concentration had an extremely significant positive correlation with pH, and was significantly positively correlated with turbidity. Some research showed that pH is an important factor that affects the morphology of quinolone antibiotics^62^. pH can affect the photodegradation process of antibiotics and is also an important parameter reflecting the trophic characteristics of water^63^. The elements nitrogen (N) and phosphorus (P) are the main factors causing reservoir eutrophication, and most of N and P compounds in the reservoir area are from domestic sewage or aquaculture wastewater^64^. Therefore, the results indicated that NOR and CIP were mainly stemmed from domestic sewage and aquaculture wastewater.

On the other hand, the adsorption behavior of pollutants in sediments is affected by many factors in the microenvironment. The physical and chemical properties of sediments and the molecular structure of pollutant compounds have impacts on the adsorption behavior of pollutants^33,65^, thus affecting the antibiotic concentrations in sediments. In this study, NOR was positively correlated with TP and TOC while OFX and ENR were significantly correlated with TN and TP respectively, which was similar to that of Ren et al.^40^. The S7 sampling site was the highest in terms of TP content and also the highest levels of NOR and ENR. S3 had the lowest level of concentration for TN and OFX. These demonstrated that there is a relationship between the concentration of quinolone antibiotics and the basic parameters of sediments. The TP in the Qingshitan reservoir area is mainly derived from the feedstuffs and the residue of the feeding animals^20^. P is not only a nutrient but also a constant mineral element, necessary for animals. TP is an indicator that reflects the level of phosphorus in the feed. TP and quinolone antibiotics are easily adsorbed in particles and sediments^60^. TP has a complex structure and contained a rich variety of phosphorus forms. The group in TP structure may be coupled with the group in quinolones antibiotic structure, strengthening the adsorption of quinolones on sediments^40^. Therefore, NOR and ENR mainly come from the aquaculture farms and livestock pastures around the reservoir area. Wastewater containing P sourced from fertilizers used for irrigation as well as from sewage containing washing powder used by residents that both will be discharged into the reservoir area. This will certainly affect the adsorption of quinolones in sediments. The higher contents of TP and TN in the sediments also reflect the high degrees of eutrophication. Therefore, it indirectly indicates that the eutrophication of water may also be accompanied by contamination problems with antibiotics^40^. The organic matter contained in sediment is complex and diverse besides, antibiotics are rapidly absorbed after entering the water, indicating that TOC has a certain influence on the contamination of antibiotics in sediments.

Comparing these two environments (water and sediment), the concentration of quinolone antibiotics in the sediment is higher than in the water. The highest concentration in the sediment was NOR, and the lowest was OFX. However, the highest concentration in water was OFX, and the lowest was NOR, which may be related to the K_d_ (adsorption constant) of quinolone antibiotics. K_d_ is commonly used to indicate the adsorption capacity of antibiotics in soil/sediment. The higher K_d_ of antibiotic is, the easier it will be adsorbed in soil/sediment. Some studies have shown that the quinolone antibiotics have high K_d_ (up to 5,000 L/kg), weak migration ability, and easy to be adsorbed on soil/sediment. Among them, the K_d_ of NOR is the highest so it is easier to be adsorbed in sediment while OFX has the lowest K_d_ (OFX 309 L/kg, NOR 506 L/kg, CIP 427 L/kg, ENR 496 L/kg).

### Ecological risk assessment in water and Sediment

The ecological risk assessment of antibiotics in water is shown in Table 7. The RQ of OFX in east and west lake were all higher than 1, indicating that OFX is at a high-risk level in the Qingshitan reservoir. The RQ of NOR, CIP, and ENR were both less than 1 and higher than 0.1, indicating that NOR, CIP, and ENR have a middle-risk level in the Qingshitan reservoir. Although the NOR, CIP and ENR concentrations are detected at low levels, there are still higher risks in ecological risk assessment, showing that these antibiotics also harm biological organisms in the water at low concentrations, and should be paid attention to.

**Table 7:** Ecological risk assessment of water in Qingshitan reservoir.

Analytes	Sensitive species	PNEC_water_/(ng/L)	RQ of East Lake	RQ of West Lake	References
OFX	*Pseudokirchneriella subcapitata*	1.13	6.89	10.94	^35^
NOR	*Microcystis aeruginosa*	1.6	0.80	0.89	^36^
CIP	*Microcystis aeruginosa*	5	0.16	0.16	^35^
ENR	*Vibrio fischeri*	28.8	0.30	0.32	^35^

As for the sediment, the RQ of the four quinolones in the east and west lake area were all higher than 1 (Table 8), indicating that the four quinolones antibiotics had a high risk for the sediment environment of Qingshitan reservoir. Most of them are excreted as prototypes and enter the environment through various means. Antibiotics and their degradation products can maintain long-term activity in the soil environment. In addition, they can inhibit the growth of certain microorganisms in water or sediments and even directly kill some microorganisms, which affect the composition of microbial communities in the environment. At the same time, the presence of antibiotics may also lead to the emergence of antibiotic-resistant bacteria^66^. The residual antibiotics in the sediments, as a possible source of damage, have high potential risks whether they are directly affecting the water environment or ultimately affecting humans through the food chain.

**Table 8:** Ecological risk assessment of sediments in Qingshitan reservoir.

Analytes	Sensitive species	K_ow_	PNEC_water_/(ng/L)	PNEC_sediment_ (ng/kg)	RQ of East Lake	RQ of West Lake	References
OFX	*Pseudokirchneriella subcapitata*	0.466	1.13	157.25	> 1	> 1	^35^
NOR	*Microcystis aeruginosa*	0.133	1.6	101.95	> 1	> 1	^36^
CIP	*Microcystis aeruginosa*	2.51	5	1990.00	> 1	> 1	^35^
ENR	*Vibrio fischeri*	4.45	28.8	16,347.15	> 1	> 1	^35^

In aquatic ecosystems, most antibiotics usually exist in the form of mixtures, which will increase the harmful effects on the environment. At present, the ecological risk assessment of antibiotic pollutants is mainly based on the toxic effect of a single compound and does not consider compound pollution, so it cannot reflect the actual risk level of aquatic ecosystem better, but this evaluation system still can be for reference^67^.

### Health risk assessment in water and fish

The range of health risk index of water for each sampling point in this study was 1.66 × 10^−6^ to 2.25 × 10^−6^, which was in the order of magnitude of 10^−6^ (Table 9). Many scholars have studied the health risks of antibiotics in drinking water sources (lakes, reservoirs), but currently, there is no standard to judge the health risks of antibiotics. Our result was similar to those reported by^68^ and^69^ (health risk assessment scale for quinolone antibiotics was on the order of 10^−6^). Compared with the thresholds for water quality, health risks of drinking water sources set by different international agencies (Table 10). The health risk index of each sampling point of Qingshitan reservoir was higher than the negligible risk and was close to the acceptable maximum risk. Residues of quinolone antibiotics may cause harm to human health.

**Table 9:** R_QH_ (risk quotient) of water of all 15 sampling sites of Qingshitan reservoir.

Sampling point	R_QH_
S1	1.71 × 10^−6^
S2	1.66 × 10^−6^
S3	2.21 × 10^−6^
S4	2.25 × 10^−6^
S5	1.87 × 10^−6^
S6	1.64 × 10^−6^
S7	1.82 × 10^−6^
S8	1.98 × 10^−6^
S9	1.79 × 10^−6^
S10	1.69 × 10^−6^
S11	2.22 × 10^−6^
S12	1.85 × 10^−6^
S13	1.82 × 10^−6^
S14	1.82 × 10^−6^
S15	1.77 × 10^−6^

**Table 10:** The maximal acceptable and neglectable risk level of water by foreign organizations.

International institutions	Acceptable maximum risk/a^−1^	Negligible risk/a^−1^	Note
Swedish Environmental Protection Agency	1 × 10^−6^	–	Chemical pollutant
Netherlands Environmental Protection Agency	1 × 10^−6^	1 × 10^−8^	Chemical pollutant
The Royal Society	1 × 10^−6^	1 × 10^−7^	–
United States Environmental Protection Agency	1 × 10^−4^	–	–
National Radiological Protection Board	5 × 10^−5^	–	Radiation

As for fish, the evaluation of antibiotic residues and health risks in protein foods from animal flesh has attracted attention from consumers^30^. China's ministry of agriculture has announced that the maximum residue limit (MRL) for enrofloxacin of muscle in aquatic products is 100 μg/kg^70^. In this sample collection, only the ENR residue of *H.leacisculus* in the west lake was higher than 100 μg/kg and the ENR concentrations of other fishes were all below 100 μg/kg. However, compared with foreign standards, there is still a large gap. For example, the residual limit of enrofloxacin and ciprofloxacin is 30 μg/kg in the United States and the European Union.

The Food and Agriculture Organization of the United Nations (FAO) and the World Health Organization (WHO) stipulate that the allowable daily intake (ADI) of ENR is 2 μg/kg. This article uses ENR residues and adult weight (60 kg) to evaluate the estimated daily intake (EDI) of fish in the Qingshitan reservoir. According to the "Outline for Food and Nutrition Development in China (2014–2020)" issued by the General Office of the State Council^71^, 49.3 g of aquatic products are consumed per person per day. The EDI of all fish samples was lower than 2 μg/kg (Table 11), indicating that the safety of fish collected in the Qingshitan reservoir is high. In addition to fish, people also consume other foods that have been detected with antibiotic residues^72,73^. A daily dose of such harmful intakes will affect public health in the long term, therefore, relevant departments should strengthen control over antibiotic drug administrations.

**Table 11:** EDI contribution of ENR in fish of Qingshitan reservoir in μg/kg.

Fish	ENR/(μg/kg)	EDI/(μg/kg)
*Carassius auratus*	44.58	0.036
*Cyprinus carpio*	14.21	0.011
*Pelteobagrus fulvidraco*	6.73	0.005
*Sinibrama macrops*	8.87	0.007
*Hemiculter leucisculus*	102.87	0.084
*Ctenopharyngodon idellus*	47.43	0.039
*Silurus asotus*	17.92	0.014

## Conclusion

The detection frequency of OFX, NOR, CIP and ENR in water, sediment and fish was 100%. OFX was observed as the highest concentration in water. NOR is more easily adsorbed in sediment than other quinolone antibiotics, while the highest OFX concentration was detected in fish. pH and turbidity in water influenced the residue and distribution of NOR, while TP impacted CIP. TP and TOC in sediment influenced the residue and distribution of NOR, while TN and TP impacted OFX and ENR respectively. The antibiotic concentration in fish was related to its species, feeding habits, and inhabited water layer. Contaminants accumulate along with the food chain and the higher the consumer level, the higher concentration of pollutants were detected at higher trophic levels. The antibiotic pollution of the reservoir cannot be ignored and long-term monitoring of antibiotics’ residues is required. Furthermore, antibiotic pollution control policy and technology need to be applied to protect the drinking water source in this area.

## Appendix

See Table 12.

**Table 12:** Recovery of four antibiotics in fish and sediment of Qingshitan reservoir.

Sample	Concentration	Recovery ± SD %^c^			
		OFX	NOR	CIP	ENR
Water	0.05 μg/mL	90.74 ± 10.79	83.17 ± 10.26	89.45 ± 12.47	79.74 ± 10.33
	0.2 μg/mL	87.52 ± 12.55	78.69 ± 8.67	89.53 ± 11.01	72.06 ± 6.64
Sediment	50 μg/kg	61.32 ± 7.84	92.39 ± 4.07	85.10 ± 16.67	76.38 ± 8.85
	500 μg/kg	67.83 ± 1.38	94.42 ± 2.70	90.07 ± 4.07	87.69 ± 5.71
Fish	10 μg/kg	93.20 ± 11.19	86.79 ± 13.19	91.07 ± 4.96	73.35 ± 0.81
	100 μg/kg	88.14 ± 14.56	83.02 ± 9.01	90.11 ± 8.79	79.73 ± 14.91
	1,000 μg/kg	89.68 ± 10.81	99.52 ± 6.36	90.82 ± 7.66	91.50 ± 4.95

^C^Mean.

*SD* standard deviation.
